# High consumption of dairy products and risk of major adverse coronary events and stroke in a Swedish population

**DOI:** 10.1017/S0007114523001939

**Published:** 2024-02-14

**Authors:** Justine Dukuzimana, Suzanne Janzi, Caroline Habberstad, Shunming Zhang, Yan Borné, Emily Sonestedt

**Affiliations:** 1 Nutritional Epidemiology, Department of Clinical Sciences Malmö, Lund University, Malmö, SE-21428, Sweden; 2 School of Public Health, Xi’an Jiaotong University Health Science Center, Xi’an, People’s Republic of China

**Keywords:** Milk, Dairy products, Coronary events, Stroke, Cohort

## Abstract

The association between the consumption of dairy products and risk of CVD has been inconsistent. There is a lack of studies in populations with high intakes of dairy products. We aimed to examine the association between intake of dairy products and risk of incident major adverse coronary events and stroke in the Swedish Malmö Diet and Cancer cohort study. We included 26 190 participants without prevalent CVD or diabetes. Dietary habits were obtained from a modified diet history, and endpoint data were extracted from registers. Over an average of 19 years of follow-up, 3633 major adverse coronary events cases and 2643 stroke cases were reported. After adjusting for potential confounders, very high intakes of non-fermented milk (>1000 g/d) compared with low intakes (<200 g/d) were associated with 35 % (95 % CI (8, 69)) higher risk of major adverse coronary events. In contrast, moderate intakes of fermented milk (100–300 g/d) were associated with a lower risk of major adverse coronary events compared with no consumption. Intakes of cheese (only in women) and butter were inversely associated with the risk of major adverse coronary events. We observed no clear associations between any of the dairy products and stroke risk. These results highlight the importance of studying different dairy foods separately. Further studies in populations with high dairy consumption are warranted.

CVD including CHD and stroke, remain the global leading cause of deaths and disability-adjusted life-years^(1)^. High blood pressure, high LDL-cholesterol, obesity, unhealthy diet and tobacco use are the top modifiable risk factors for CVD^(1)^. SFA, mainly of animal origin, is known to increase circulating LDL-cholesterol concentration^(2)^. As dairy products (i.e. milk and other dairy products) are a major source of SFA, reducing their intake, especially high-fat dairy products, has been recommended for the prevention of CVD^(3,4)^. However, dairy products have a complex mixture of nutrients and other bioactive components including specific amino acids, vitamin K, Ca, medium and odd-chain SFA and unsaturated fatty acids, which might influence coronary health and cardiometabolic pathways^(5)^. Additionally, the processing methods result in diverse types of dairy products, such as fermented dairy products (e.g. fermented milk and cheese) and non-fermented milk, which could have different effects on coronary health^(6)^.

Numerous studies have examined the association between the consumption of dairy products and the risk of CVD. Many studies have shown inverse associations or no associations, while others reported positive associations between intake of milk or other dairy products and increased risk of CVD or CVD mortality^(3,6–11)^. Thus, the association between the consumption of dairy products and the risk of CVD remains inconsistent. In addition, very few studies have examined the risk associated with very high intakes. For example, studies examining the risk with very high intake levels (i.e. more than 1 l of milk/d) are lacking. We have previously found inverse associations across quintiles for fermented milk and cheese (only in women) and risk of CVD during an average of 12 years of follow-up within the Swedish Malmö Diet and Cancer (MDC) cohort^(12)^. We now aimed to examine the association between specific types of milk (non-fermented and fermented milk), other dairy products (cheese, butter and cream) and the risk of major adverse coronary events, CHD and stroke (total and subtypes) with an additional 7 years of follow-up and with a focus on more extreme intakes (e.g. consumption of more than 1 l of milk daily).

## Methods and materials

### Selection criteria and data collection

This study included participants from the MDC cohort. The MDC is a Swedish population-based cohort study with baseline examination conducted in 1991–1996 from the source population comprising 74 138 women and men born in 1923–1950 and living in the city of Malmö. The MDC study has been previously detailed^(13)^. To participate in the MDC study, the individuals must be fluent in Swedish and had no mental incapacity. This study was conducted according to the guidelines laid down in the Declaration of Helsinki. The study was approved by the Ethical Review Board at the Medical Faculty at Lund University (LU 51-90) and the study participants signed written informed consent forms before participating in this study.

**Fig. 1.**
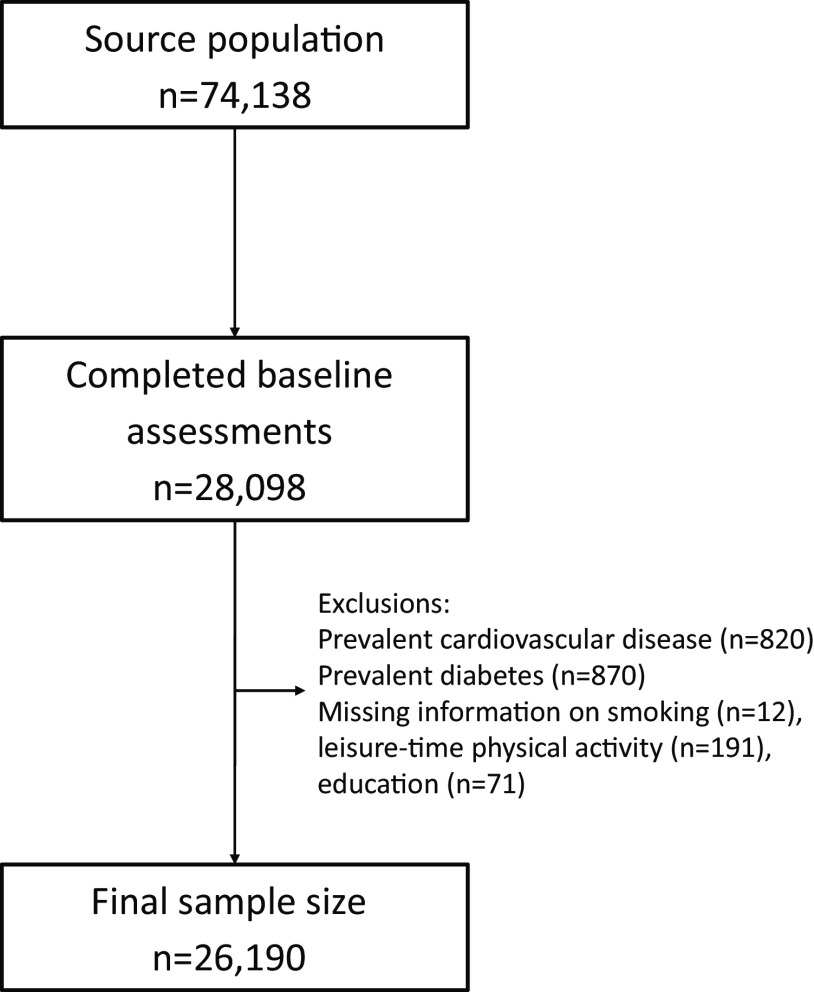
Flow chart of sample selection from the Malmö Diet and Cancer study.

During the recruitment period, the participants visited the study centre twice. On the first visit, each participant measured anthropometrics and answered a detailed self-administered questionnaire that covered smoking status, physical activity level, socio-economic status, demographic factors, educational level, medication and dietary supplement use. Additionally, the participants were instructed to record their dietary intake. On the second visit, a diet interview was conducted. Complete baseline examinations (i.e. lifestyle questionnaires, dietary assessment and anthropometric measurements) were obtained from 28 098 participants. After excluding individuals with prevalent CVD (*n* 820), prevalent diabetes or diabetes medication use (*n* 870), incomplete information on leisure-time physical activity (*n* 191), smoking status (*n* 12) and educational level (*n* 71), 26 190 individuals remained (9947 men and 16 243 women) (Fig. 1).

### Diet assessment

Information on dietary habits was obtained from the diet history method^(14,15)^ comprised of a dietary questionnaire, a 7-d food record and a diet interview. In the 7-d food record, the participants recorded their intakes of lunch and dinner meals, cold beverages and supplements. The dietary questionnaire recorded the frequency and portion size of 168 items not covered in the food record (e.g. breakfast snacks and hot drinks) during the year prior to the study. In the 7-d food record, the amount of both non-fermented milk and fermented milk (i.e. yogurt and sour milk) consumed as drinks was recorded according to the fat content (low: ≤0·5 % fat, medium: 0·6–2·4 % fat and high, 2·5–7 % fat), and other dairy products in the cooked meals were collected. Other intakes of dairy products not covered by the food records such as milk in tea, milk-based spread on bread, milk in porridge and cereals, chocolate milk, yogurt, cream in coffee and on fruits compote, butter on bread and cheese not in hot meals were recorded in the dietary questionnaire. During the 1-h diet interview, which was shortened to 45 min interview in September 1994, information was collected on serving sizes and preparation methods of foods recorded in the 7-d food record. The average daily intake of non-fermented milk, fermented milk and other dairy products was calculated as a sum of the intake from the food record and the dietary questionnaire. We categorised the intake of dairy products (g/d) in groups to get as wide intake ranges as possible but still with enough number of participants in each group. The cut-offs have been used in a previous publication^(16)^. Non-fermented milk was categorised into 0–200, 200–400, 400–600, 600–800, 800–1000 and above 1000 g/d; fermented milk was categorised into 0, 0–100, 100–200, 200–300 and above 300 g/d; cheese was categorised into 0–20, 20–40, 40–60, 60–80, 80–100 and >100 g/d; cream intake was categorised into 0–10, 10–20, 20–30, 30–40, 40–50 and >50 g/d and butter intake was categorised into 0, 0–10, 10–20, 20–30, 30–40, 40–50 and >50 g/d. Total energy intake was recorded in MJ/d and fibre density was recorded in g/4.18 MJ. Coffee, meat (processed and unprocessed), vegetables and fruits and sugar-sweetened beverages were recorded in grams per day.

### Other covariates

BMI was calculated dividing measured body weight in kilograms by measured height in metres squared (kg/m^2^). Lifestyle covariates were collected through a self-administered questionnaire. Smoking status was categorised as never smokers, former smokers and current smokers. Alcohol consumption was categorised into sex-specific quintiles, while people who reported neither alcohol intake in the 7 d food record nor for the previous year were classified as zero consumers. The adapted Minnesota physical activity instrument^(17)^ was used to collect information on time spent on seventeen different leisure-time physical activities. The metabolic equivalent of the task in hours per week (MET h/week) was calculated, and the participants were categorised into five groups based on their total MET h/week: below 7·5, 7·5–15, 15–25, 25–50 and above 50. Education was categorised into five groups: elementary school, primary and secondary school, upper secondary school, further education without a degree and education with a university degree. Individuals reporting a substantial change in dietary habits in the questionnaire were classified as diet changers (*n* 5684) with twenty-five individuals not having answered this question^(18)^. Individuals with a potential misreporting of energy (*n* 3995 under-reporters and 811 over-reporters) were defined as having a reported energy intake to BMR outside of the 95 % CI of physical activity level (estimated from information on physical activity at work, during leisure time, household work, estimated sleeping hours, self-care and passive time^(19)^).

### Outcome assessment

The study participants were followed from entry until the diagnosis of major adverse coronary events or stroke, death, emigration or end of follow-up on 31 December 2016, whichever came first. Through the personal identity number, the endpoints were extracted from the Swedish Hospital Discharger Register and the cause of death register. Major adverse coronary events included CHD, coronary artery bypass graft surgery and percutaneous coronary artery intervention. For CHD, we used the International Classification of Diseases (ICD) 9th revision codes 410–414 or other corresponding codes, which included fatal and non-fatal myocardial infarction or death due to ischaemic heart disease. For stroke, we used the ICD-9 codes 430 (subarachnoid haemorrhage), 431 (intracerebral haemorrhage), 434 (cerebral infarction/ischaemic stroke) and 436 (unspecified stroke). Validation of stroke diagnoses that occurred at Malmö University hospital was conducted until 2010 by review of medical records and in most cases also through patient interview^(20)^. Autopsy, computed tomography or MRI was used to diagnose ischaemic stroke and/or exclude haemorrhage or non-vascular disease. Stroke was classified as unspecified if neither imaging nor autopsy was executed. Ischaemic and haemorrhagic (i.e., subarachnoid and intracerebral) stroke were examined separately.

### Statistical analysis

Cox regression proportional hazards models were applied to estimate the hazard ratios (HR) and 95 % CI for developing major adverse coronary events, CHD and stroke associated with categories of dairy consumption. Time of follow-up was used as the underlying time variable. Several risk factors were identified as potential confounders for the association between dairy consumption and the risk of cardiovascular outcomes. To reduce the bias by potential confounders, model 1 (the basic model) was adjusted for age (continuous), sex (categorical), diet interview method (categorical), season (categorical) and total energy intake (continuous). Model 2 was further adjusted for alcohol (categorical), smoking (categorical), education (categorical), leisure-time physical activity (categorical) and diet intake (continuous) of fibre, vegetables, fruits, meat, sugar-sweetened beverages and coffee. In model 3 (full model), we additionally adjusted for BMI (continuous) because BMI can also be considered a mediating factor and it is important to compare the risk estimates with and without BMI in the model. *P*_for trend_ was calculated to assess the linear association between milk and other dairy consumption and the cardiovascular outcomes. We further used the restricted cubic splines with four knots, placed according to Harrell’s recommended percentiles (5 %, 35 %, 65 % and 95 %) with reference at 0 g, to assess the shape and potential trend of linear association between dairy consumption and cardiovascular outcomes.

To test the robustness of the results, in sensitivity analysis we excluded all participants who potentially misreported energy intake (both under and over reporters)^(19)^, and those who indicated that they had changed their diet habits before baseline examinations^(18)^ (*n* 9259). We also run sensitivity analyses to examine the effect of adjusting for energy intake using the residual method and the nutrient density method. The energy-adjusted intakes were standardised according to the median energy intake in this population (9·55 MJ). Participants were thereafter categorised using the same cut-offs based on their energy-adjusted intake values. We examined interaction with sex, BMI or leisure-time physical activity by introducing a multiplicative factor between these variables and the dietary variables in the full model. For statistically significant interactions, we further performed stratified analyses by sex, BMI groups (below and above 25 kg/m^2^) or leisure-time activity groups (below and above 25 MET h/week). Statistical analyses were performed using Statistical Package for the Social Sciences software version 27 (SPSS version 27.0; IBM Corp.), and Survival R package version 3.2-13 was used for restricted cubic splines. The two-sided *P*-value <0·05 was considered statistically significant.

## Results

### Characteristics of the study population

Table 1 shows the characteristics of 26 190 participants (62 % women; mean age 57·8 (sd 7·6), range: 44–74 years) across categories of non-fermented and fermented milk intakes. Higher compared with lower consumers of non-fermented milk tended to have higher BMI, high intakes of energy, meat, soft drink and coffee, higher frequency of smokers and low intakes of fruits and vegetables, whereas high consumers of fermented milk tended to have low intakes of meat, low frequency of smokers and high intake of fruits and vegetables.

**Table 1.** Baseline characteristics of the study participants across intakes of non-fermented and fermented milk (Numbers and percentages; mean values and standard deviations)

Characteristics	Non-fermented milk (g/d)											
	0–200 (*n* 11 655)		200–400 (*n* 8011)		400–600 (*n* 4155)		600–800 (*n* 1482)		800–1000 (*n* 495)		>1000 (*n* 392)	
	*n*	%	*n*	%	*n*	%	*n*	%	*n*	%	*n*	%
Women	7515	64·5	5174	64·6	2491	60·0	750	50·6	217	43·8	96	24·5
	Mean	sd	Mean	sd	Mean	sd	Mean	sd	Mean	sd	Mean	sd
Age (years)	57·2	7·4	58·3	7·6	58·6	7·7	58·3	7·6	57·5	7·6	57·0	7·6
BMI (kg/m^2^)	25·3	3·8	25·7	3·9	25·9	3·9	26·0	4·2	26·4	4·3	26·5	4·1
	%		%		%		%		%		%	
Smokers	26·4		27·7		29·8		34·0		35·2		51·8	
Zero consumers of alcohol	4·2		6·0		8·9		9·0		11·9		12·5	
Elementary education	36·0		42·6		46·7		52·1		47·7		55·6	
Low physical activity (<7·5 MET/week)	9·3		8·8		9·7		10·1		11·5		13·8	
Dietary intake												
	Mean	sd	Mean	sd	Mean	sd	Mean	sd	Mean	sd	Mean	sd
Energy intake (MJ/d)	8.96	2.52	9.49	2.52	10.09	2.65	11.11	2.92	11.87	3.36	13.38	3.87
Meat (g/d)	129·1	63·4	131·4	59·0	136·1	62·8	146·3	68·4	150·3	74·7	179·9	95·7
Fibre density (g/4.18 MJ)	9·7	2·9	9·2	2·5	8·9	2·4	8·4	2·4	8·1	2·4	7·1	2·3
Vegetables and fruits (g/d)	383·8	194	370·8	172	375·9	179	369·3	188	359·4	194	317·1	188
Soft drinks (g/d)	71·8	141	73·3	139	86·6	149	96·1	166	99·3	164	124·3	254
Coffee (g/d)	511·7	393	511·8	366	527·0	392	545·6	418	576·59	457	712·3	676
Fermented milk (g/d)												
	0 (*n* 9102)		0–100 (*n* 7940)		100–200 (*n* 5728)		200–300 (*n* 2364)		>300 (*n* 1056)			
	*n*	%	*n*	%	*n*	%	*n*	%	*n*	%		
Women	4684	51·5	5568	70·1	3902	68·1	1494	63·2	595	56·3		
	Mean	sd	Mean	sd	Mean	sd	Mean	sd	Mean	sd		
Age (years)	58·4	7·5	57·3	7·6	57·8	7·6	57·8	7·4	57·3	7·5		
BMI (kg/m^2^)	25·8	4·0	25·6	3·9	25·5	3·8	25·2	3·5	25·2	3·6		
	%		%		%		%		%			
Smokers	33·4		27·4		24·1		24·1		24·4			
Zero consumers of alcohol	7·3		5·5		5·0		5·3		7·0			
Elementary education	49·1		39·0		36·5		34·0		29·8			
Low physical activity (<7·5 MET/week)	12·2		8·5		7·3		7·1		7·9			
Dietary intake												
	Mean	sd	Mean	sd	Mean	sd	Mean	sd	Mean	sd		
Energy intake (MJ/d)	9.57	2.89	9.29	2.60	9.50	2.57	9.84	2.63	10.73	3.00		
Meat (g/d)	144·1	68·7	129·0	60·1	125·5	58·5	125·1	60·3	126·6	64·5		
Fibre density (g/4.18 MJ)	8·8	2·8	9·4	2·6	9·6	2·5	9·7	2·7	9·7	2·9		
Vegetables and fruits (g/d)	339·0	183	381·4	178	398·9	177	417·6	186	444·7	223		
Soft drinks (g/d)	83·7	165	77·6	138	67·3	121	71·9	142	86·0	171		
Coffee (g/d)	537·5	416	511·3	386	510·5	371	506·7	377	523·7	417		

MET, metabolic equivalent time.

### Milk and other dairy consumption and the risk of major adverse coronary events and stroke

**Fig. 2.**
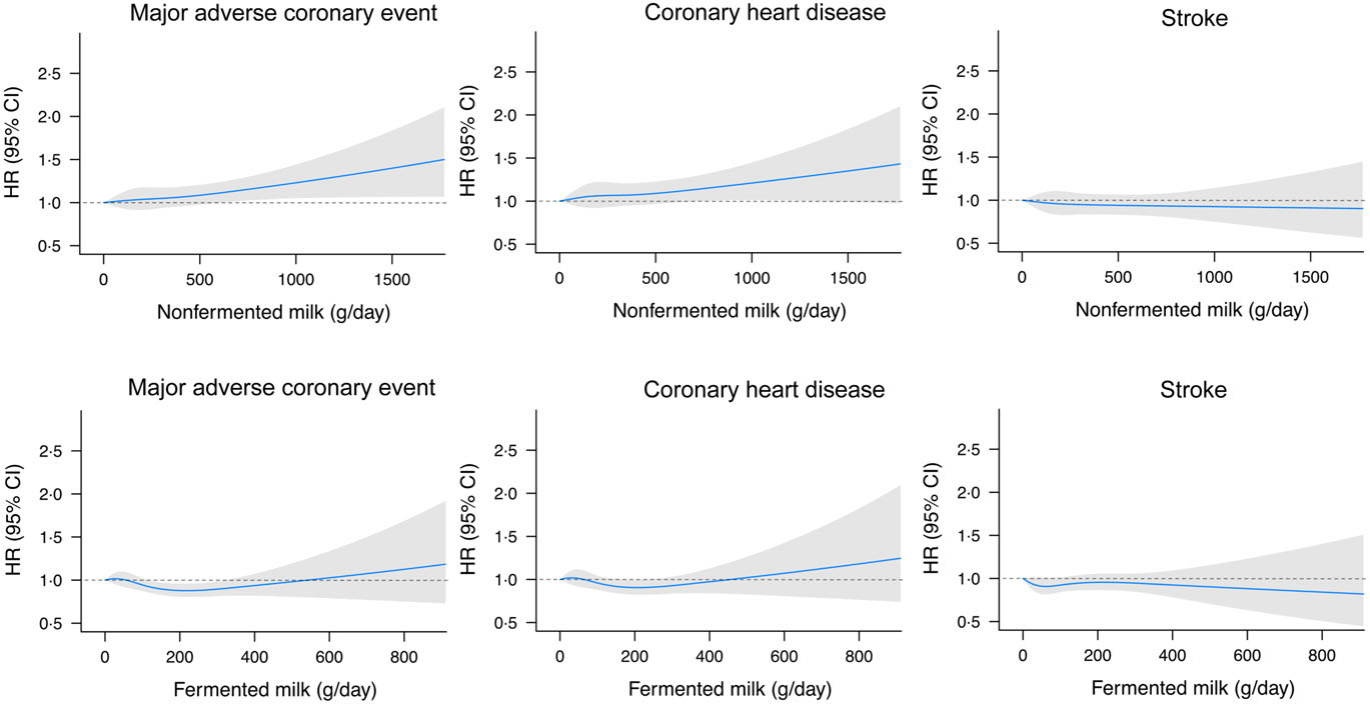
Restricted cubic splines for the associations between milk intakes and risk of major coronary events, CHD and stroke with 0 g/d as the reference value. The HR and 95 % CI were adjusted for age, sex, assessment method, season and energy, alcohol, smoking, education, physical activity, fibre, vegetable and fruits, meat, soft drinks, coffee and BMI.

Over an average of 19 years (maximum = 26 years), and 501 632 persons-years of follow-up, a total of 3633 (13·9 %) incident cases of major adverse coronary events (including 2905 cases with CHD without percutaneous coronary artery intervention or coronary artery bypass graft surgery) and 2643 (10·1 %) stroke cases were reported (including 2155 ischaemic stroke and 445 haemorrhagic stroke). Very high (>1000 g/d) compared with low (<200 g/d) consumption of non-fermented milk was associated with a 35 % (95 % CI (8, 69)) higher risk of major adverse coronary events and 30 % (95 % CI (1, 68)) higher risk of CHD in the full model (Table 2), with a dose–response association observed (Fig. 2). We observed no significant association between non-fermented milk and total stroke.

**Table 2.** Association between non-fermented milk intakes and risk of major adverse coronary events and stroke (Hazard ratios and 95 % confidence intervals)

Outcomes	Non-fermented milk (g/d)													*P*_trend_
	0–200 (*n* 11 655)	200–400 (*n* 8011)		400–600 (*n* 4155)		600–800 (*n* 1482)		800–1000 (*n* 495)		>1000 (*n* 392)		Per 100 g/d		
		HR	95 % CI	HR	95 % CI	HR	95 % CI	HR	95 % CI	HR	95 % CI	HR	95 % CI	
Major adverse coronary events	1462	1122		633		247		78		91				
Person-years	227 480	153 596		77 961		26 871		9164		6559				
Model 1*	1·00	1·08	1·00, 1·17	1·15	1·05, 1·27	1·27	1·10, 1·45	1·17	0·93, 1·47	1·85	1·48, 2·30	1·04	1·03, 1·06	<0·001
Model 2†	1·00	1·05	0·97, 1·14	1·09	0·99, 1·20	1·14	0·99, 1·31	1·05	0·83, 1·33	1·45	1·16, 1·82	1·03	1·01, 1·04	<0·001
Model 3‡	1·00	1·03	0·96, 1·12	1·06	0·96, 1·16	1·10	0·95, 1·26	1·00	0·79, 1·27	1·35	1·08, 1·69	1·02	1·01, 1·03	0·005
CHD	1149	916		513		190		66		71				
Person-years	230 567	155 747		79 261		27 584		9326		6791				
Model 1	1·00	1·11	1·02, 1·21	1·17	1·06, 1·30	1·22	1·04, 1·42	1·27	0·99, 1·64	1·85	1·45, 2·37	1·05	1·03, 1·06	<0·001
Model 2	1·00	1·07	0·98, 1·17	1·09	0·98, 1·21	1·06	0·90, 1·24	1·10	0·86, 1·42	1·40	1·09, 1·80	1·02	1·01, 1·04	0·003
Model 3	1·00	1·05	0·96, 1·15	1·06	0·95, 1·18	1·02	0·87, 1·20	1·05	0·82, 1·36	1·30	1·01, 1·68	1·02	1·00, 1·03	0·03
Total stroke	1113	839		452		146		55		38				
Person-years	230 192	155 913		79 468		27 839		9326		6888				
Model 1	1·00	1·02	0·93, 1·12	1·04	0·93, 1·17	0·97	0·82, 1·16	1·15	0·88, 1·52	1·08	0·78, 1·51	1·01	0·99, 1·02	0·41
Model 2	1·00	1·00	0·91, 1·10	1·01	0·90, 1·13	0·90	0·75, 1·08	1·06	0·81, 1·40	0·91	0·65, 1·27	1·00	0·98, 1·01	0·65
Model 3	1·00	0·99	0·90, 1·09	0·99	0·88, 1·11	0·88	0·74, 1·06	1·04	0·79, 1·37	0·88	0·63, 1·23	0·99	0·98, 1·01	0·40
Ischaemic stroke	937	665		356		123		46		28				
Model 1	1·00	0·96	0·87, 1·06	0·97	0·86, 1·10	0·97	0·80, 1·17	1·13	0·84, 1·53	0·94	0·64, 1·37	1·00	0·98, 1·02	0·98
Model 2	1·00	0·93	0·84, 1·03	0·93	0·82, 1·05	0·89	0·73, 1·08	1·03	0·76, 1·40	0·77	0·53, 1·14	0·99	0·97, 1·01	0·24
Model 3	1·00	0·92	0·83, 1·02	0·91	0·80, 1·03	0·87	0·71, 1·06	0·99	0·73, 1·35	0·74	0·50, 1·09	0·98	0·96, 1·00	0·10
Haemorrhagic stroke	158	162		84		23		9		9				
Model 1	1·00	1·42	1·14, 1·77	1·41	1·07, 1·84	1·12	0·72, 1·75	1·38	0·70, 2·73	1·92	0·96, 3·84	1·04	1·00, 1·08	0·06
Model 2	1·00	1·42	1·14, 1·78	1·39	1·05, 1·83	1·04	0·66, 1·66	1·35	0·68, 2·69	1·69	0·84, 3·42	1·03	0·99, 1·07	0·16
Model 3	1·00	1·43	1·14, 1·79	1·40	1·06, 1·84	1·06	0·67, 1·67	1·37	0·69, 2·73	1·72	0·85, 3·47	1·03	0·99, 1·08	0·14

* Model 1: adjusted for age, sex, diet assessment method, season and energy.

† Model 2: adjusted for age, sex, diet assessment method, season, energy, alcohol, smoking, education, physical activity, fibre, vegetable and fruits, meat, soft drinks and coffee.

‡ Model 3: adjusted for age, sex, diet assessment method, season, energy, alcohol, smoking, education, physical activity, fibre, vegetable and fruits, meat, soft drinks, coffee and BMI.

U-shaped associations were observed for fermented milk and major adverse coronary events and CHD (Fig. 2). Moderate intakes of fermented milk (100–300 g/d) were associated with a lower risk of major adverse coronary events compared with zero-consumtion (Table 3). For CHD, intakes between 200 and 300 g/d were associated with 16 % (95 % CI (3, 28)) decreased risk. For total stroke, although intakes between 0 and 100 g/d of fermented milk were associated with a slightly decreased risk compared with no consumption, there was no clear association with higher intakes. We observed no difference depending on the stroke subtype.

**Table 3.** Association between fermented milk intakes and risk of major adverse coronary events and stroke (Hazard ratios and 95 % confidence intervals)

Outcomes	Fermented milk (g/d)											*P*_trend_
	0 (*n* 9102)	0–100 (*n* 7940)		100–200 (*n* 5728)		200–300 (*n* 2364)		>300 (*n* 1056)		Per 100 g/d		
		HR	95 % CI	HR	95 % CI	HR	95 % CI	HR	95 % CI	HR	95 % CI	
Major adverse coronary events	1509	1009		695		275		145				
Person-years	167 679	154 373		112 633		46 353		20 594				
Model 1*	1·00	0·91	0·84, 0·99	0·80	0·73, 0·88	0·74	0·65, 0·84	0·85	0·72, 1·01	0·93	0·90, 0·96	<0·001
Model 2†	1·00	0·98	0·90, 1·06	0·90	0·82, 0·99	0·83	0·73, 0·95	0·97	0·82, 1·15	0·96	0·93, 1·00	0·03
Model 3‡	1·00	0·97	0·90, 1·06	0·90	0·82, 0·99	0·84	0·74, 0·96	0·98	0·82, 1·16	0·97	0·94, 1·00	0·04
CHD	1198	808		564		214		121				
Person-years	171 047	156 425		113 912		47 049		20 844				
Model 1	1·00	0·91	0·84, 1·00	0·82	0·74, 0·91	0·72	0·63, 0·84	0·91	0·75, 1·10	0·93	0·90, 0·97	<0·001
Model 2	1·00	0·99	0·91, 1·09	0·93	0·84, 1·04	0·83	0·72, 0·96	1·06	0·87, 1·28	0·98	0·94, 1·01	0·19
Model 3	1·00	0·99	0·90, 1·08	0·94	0·84, 1·04	0·84	0·72, 0·97	1·06	0·88, 1·28	0·98	0·94, 1·02	0·25
Total stroke	1037	712		563		240		91				
Person-years	171 406	156 591		113 667		46 848		21 115				
Model 1	1·00	0·85	0·77, 0·93	0·87	0·78, 0·97	0·89	0·77, 1·02	0·75	0·61, 0·93	0·95	0·92, 0·99	0·007
Model 2	1·00	0·89	0·81, 0·99	0·95	0·86, 1·06	0·98	0·85, 1·13	0·83	0·67, 1·03	0·98	0·94, 1·02	0·29
Model 3	1·00	0·89	0·81, 0·98	0·95	0·86, 1·06	0·98	0·85, 1·13	0·83	0·67, 1·04	0·98	0·95, 1·02	0·33
Ischaemic stroke	852	573		452		202		76				
Model 1	1·00	0·84	0·75, 0·93	0·86	0·76, 0·96	0·92	0·79, 1·07	0·77	0·61, 0·97	0·96	0·92, 1·00	0·05
Model 2	1·00	0·89	0·80, 0·99	0·94	0·84, 1·06	1·02	0·87, 1·19	0·86	0·68, 1·09	0·99	0·95, 1·03	0·72
Model 3	1·00	0·89	0·80, 0·99	0·95	0·84, 1·06	1·02	0·88, 1·20	0·86	0·68, 1·10	0·99	0·95, 1·04	0·79
Haemorrhagic stroke	165	128		102		36		14				
Model 1	1·00	0·90	0·72, 1·14	0·95	0·74, 1·22	0·81	0·56, 1·16	0·70	0·40, 1·21	0·92	0·84, 1·01	0·09
Model 2	1·00	0·93	0·74, 1·18	1·00	0·78, 1·29	0·85	0·59, 1·23	0·74	0·42, 1·28	0·94	0·85, 1·03	0·20
Model 3	1·00	0·93	0·74, 1·18	1·00	0·78, 1·29	0·85	0·59, 1·23	0·73	0·43, 1·28	0·94	0·85, 1·03	0·19

* Model 1: adjusted for age, sex, diet assessment method, season and energy.

† Model 2: adjusted for age, sex, diet assessment method, season, energy, alcohol, smoking, education, physical activity, fibre, vegetable and fruits, meat, soft drinks and coffee.

‡ Model 3: adjusted for age, sex, diet assessment method, season, energy, alcohol, smoking, education, physical activity, fibre, vegetable and fruits, meat, soft drinks, coffee and BMI.

**Fig. 3.**
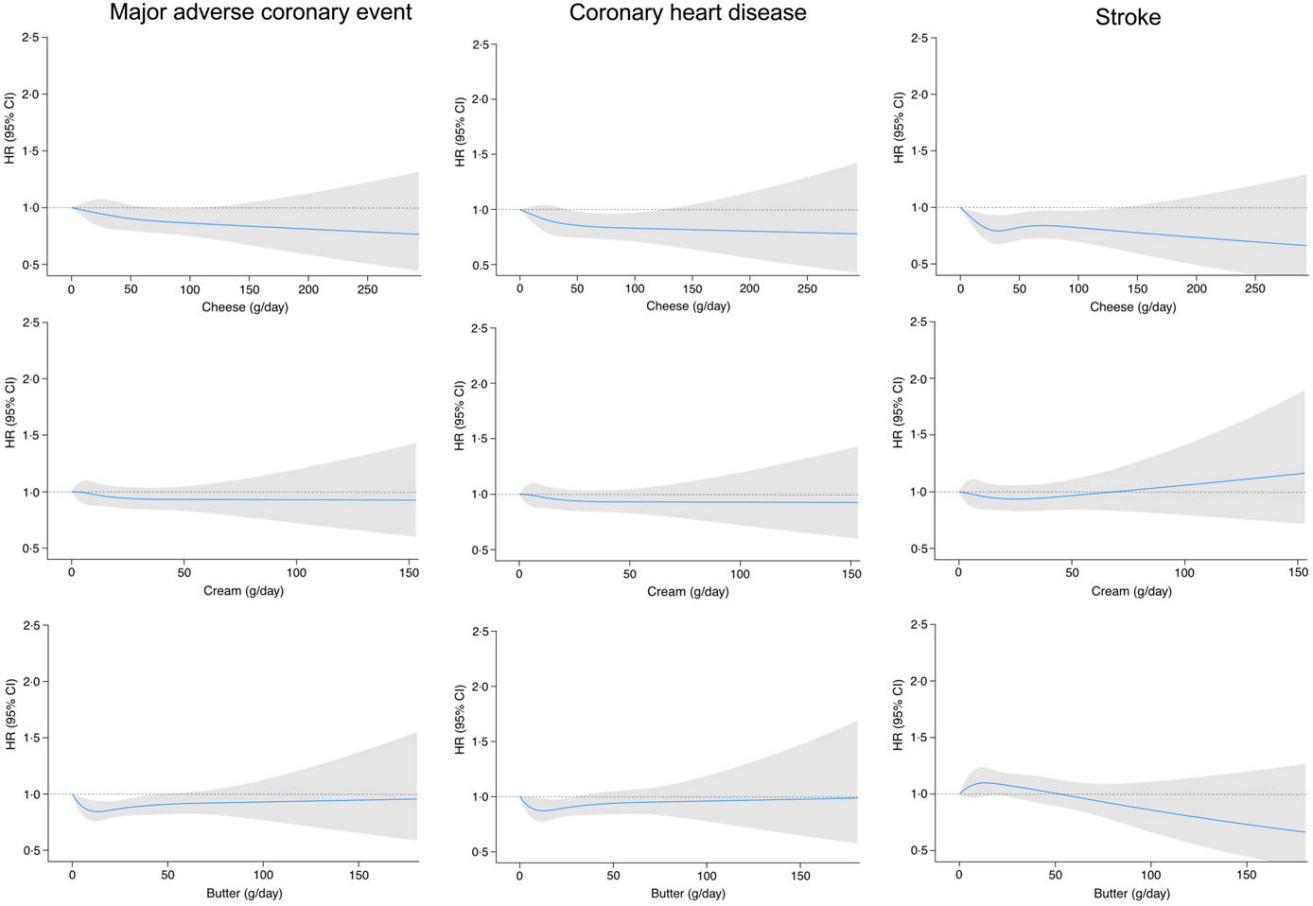
Restricted cubic splines for the associations between intakes of cheese, cream and butter and risk of major coronary events, CHD and stroke. The HR and 95 % CI were adjusted for age, sex, assessment method, season and energy, alcohol, smoking, education, physical activity, fibre, vegetable and fruits, meat, soft drinks, coffee and BMI.

Cheese intake was inversely associated with the risk of major adverse coronary events and CHD (*P*_trend_ = 0·04) (Table 4), with a dose–response association observed (Fig. 3). We observed no linear association between cheese intake and stroke risk. However, moderate intakes of cheese (20–40 g/d) were associated with a 10 % (95 % CI (0, 18)) lower risk of total stroke compared with low intakes (0–20 g/d). Similar risk reductions, although not statistically significant, were observed with higher intakes. We observed no significant association between cream consumption and any of the cardiovascular outcomes (online Supplementary Table 1 and Fig. 3). Butter consumption of 0–10 g/d, 10–20 g/d and above 50 g/d compared with zero consumption was associated with approximately 15 % reduced risk of major adverse coronary events, but no association was observed for stroke (*P*_trend_ = 0·82) (online Supplementary Table 2). Excluding potential misreporters of energy intake and diet changers did not substantially change the results (online Supplementary Table 3). However, the opposite direction observed for non-fermented milk with different stroke subtypes was more pronounced with a decreased risk of ischaemic stroke (*P*_trend_ = 0·02) and a tendency towards increased risk of haemorrhagic stroke (*P*_trend_ = 0·07). Adjusting the intake variables for energy using the residual model or the nutrient density model did not substantially change the results (online Supplementary Tables 4 and 5). However, there was a linear protective association between butter on major adverse coronary events.

**Table 4.** Association between cheese and risk of major adverse coronary events and stroke (Hazard ratios and 95 % confidence intervals)

Outcomes	Cheese (g/d)													
	0–20 (*n* 6211)	20–40 (*n* 8470)		40–60 (*n* 5885)		60–80 (*n* 2919)		80–100 (*n* 1469)		>100 (*n* 1236)		Per 10 g/d		*P*_trend_
		HR	95 % CI	HR	95 % CI	HR	95 % CI	HR	95 % CI	HR	95 % CI	HR	95 % CI	
Major adverse coronary events	990	1201		757		372		170		143				
Person-years	114 389	161 518		114 996		57 052		29 355		24 322				
Model 1*	1·00	0·91	0·84, 0·99	0·84	0·76, 0·93	0·87	0·77, 0·98	0·75	0·64, 0·89	0·78	0·65, 0·94	0·98	0·96, 0·99	<0·001
Model 2†	1·00	0·97	0·89, 1·05	0·92	0·83, 1·02	0·97	0·86, 1·10	0·85	0·72, 1·01	0·90	0·75, 1·09	0·99	0·98, 1·00	0·08
Model 3‡	1·00	0·97	0·89, 1·06	0·92	0·84, 1·02	0·96	0·85, 1·09	0·84	0·71, 1·00	0·88	0·73, 1·06	0·99	0·98, 1·00	0·04
CHD	825	955		589		289		137		110				
Person-years	116 261	164 167		116 599		57 946		29 637		24 668				
Model 1	1·00	0·87	0·80, 0·96	0·80	0·72, 0·89	0·84	0·73, 0·96	0·77	0·64, 0·92	0·75	0·61, 0·93	0·97	0·96, 0·99	<0·001
Model 2	1·00	0·93	0·85, 1·03	0·88	0·79, 0·99	0·95	0·82, 1·09	0·87	0·72, 1·05	0·87	0·70, 1·08	0·99	0·97, 1·00	0·08
Model 3	1·00	0·94	0·85, 1·03	0·88	0·79, 0·99	0·94	0·82, 1·08	0·86	0·71, 1·04	0·85	0·69, 1·05	0·99	0·97, 1·00	0·04
Total stroke	743	851		556		262		125		106				
Person-years	116 181	164 326		116 435		58 297		29 760		24 628				
Model 1	1·00	0·86	0·78, 0·95	0·86	0·76, 0·96	0·86	0·74, 0·99	0·81	0·67, 0·99	0·86	0·70, 1·07	0·98	0·97, 1·00	0·02
Model 2	1·00	0·90	0·82, 1·00	0·91	0·81, 1·02	0·93	0·80, 1·07	0·89	0·73, 1·08	0·95	0·76, 1·19	0·99	0·98, 1·01	0·29
Model 3	1·00	0·90	0·82, 1·00	0·91	0·81, 1·02	0·92	0·80, 1·07	0·88	0·73, 1·08	0·94	0·76, 1·17	0·99	0·98, 1·01	0·24
Ischaemic stroke	622	676		465		205		98		89				
Model 1	1·00	0·82	0·74, 0·92	0·86	0·76, 0·98	0·81	0·69, 0·96	0·77	0·62, 0·96	0·88	0·69, 1·11	0·98	0·97, 1·00	0·03
Model 2	1·00	0·86	0·77, 0·97	0·92	0·81, 1·04	0·88	0·74, 1·04	0·84	0·67, 1·05	0·98	0·77, 1·25	0·99	0·98, 1·01	0·36
Model 3	1·00	0·87	0·77, 0·97	0·92	0·81, 1·05	0·87	0·74, 1·03	0·84	0·67, 1·04	0·97	0·76, 1·23	0·99	0·98, 1·01	0·29
Haemorrhagic stroke	107	163		84		52		23		16				
Model 1	1·00	1·11	0·87, 1·42	0·85	0·64, 1·14	1·11	0·79, 1·56	0·97	0·61, 1·55	0·82	0·47, 1·42	0·98	0·95, 1·02	0·34
Model 2	1·00	1·16	0·90, 1·48	0·90	0·67, 1·21	1·18	0·83, 1·68	1·07	0·67, 1·72	0·85	0·48, 1·52	0·99	0·96, 1·03	0·61
Model 3	1·00	1·16	0·90, 1·48	0·90	0·67, 1·21	1·18	0·83, 1·21	1·07	0·67, 1·72	0·86	0·48, 1·52	0·99	0·96, 1·03	0·62

* Model 1: adjusted for age, sex, diet assessment method, season and energy.

† Model 2: adjusted for age, sex, diet assessment method, season, energy, alcohol, smoking, education, physical activity, fibre, vegetable and fruits, meat, soft drinks and coffee.

‡ Model 3: adjusted for age, sex, diet assessment method, season, energy, alcohol, smoking, education, physical activity, fibre, vegetable and fruits, meat, soft drinks, coffee and BMI.

There was a significant interaction between sex and cheese consumption on risk of major adverse coronary events (*P*_interaction_ = 0·04) and CHD (*P*_interaction_ = 0·02) (online Supplementary Table 6) with a linear association between cheese intake and lower risk of major adverse coronary events (HR_per 10 g_ = 0·97; 95 % CI (0·95, 0·99); *P*_trend_ = 0·003) and CHD (HR_per 10 g_ = 0·96; 95 % CI (0·94, 0·99); *P*_trend_ = 0·002) in females but not in males (HR_per 10 g_ = 1·00; 95 % CI (0·98, 1·01); *P*_trend_ = 0·60 and HR_per 10 g_ = 1·00; 95 % CI (0·98, 1·02); *P*_trend_ = 0·77, respectively). In addition, non-fermented was associated with a decreased risk of stroke among males (HR_per 100 g_ = 0·98; 95 % CI (0·95, 1·00); *P*_trend_ = 0·04) but not among women (HR_per 100 g_ = 1·01; 95 % CI (0·99, 1·04); *P*_trend_ = 0·32). No interaction with BMI was observed; however, non-fermented milk was associated with increased risk of major adverse coronary events and CHD among individuals with high level of leisure-time physical activity (HR_per 100 g_ = 1·03; 95 % CI (1·01, 1·05); *P*_trend_ = 0·004 and HR_per 100 g_ = 1·03; 95 % CI (1·01, 1·05); *P*_trend_ = 0·008) but not among individuals with low level of leisure-time physical activity (HR_per 100 g_ = 1·01; 95 % CI (0·99, 1·03); *P*_trend_ = 0·28 and HR_per 100 g_ = 1·00; 95 % CI (0·98, 1·03); *P*_trend_ = 0·73). There was also an interaction between cream and leisure-time physical activity for risk of stroke; however, there was no statistically significant trend in any of the groups (HR_per 10 g_ = 0·97; 95 % CI (0·94, 1·01); *P*_trend_ = 0·13 and HR_per 10 g_ = 1·02; 95 % CI (0·99, 1·05); *P*_trend_ = 0·26).

## Discussion

In this Swedish population-based cohort study, very high consumption of non-fermented milk (>1000 g/d) was associated with a 35 % increased risk of major adverse coronary events and a 30 % increased risk of CHD compared with low intakes. In contrast, moderate intakes of fermented milk were inversely associated with the risk of major adverse coronary events. High consumers of butter (>50 g/d) had a 15 % lower risk of major adverse coronary events compared with low consumers (0 g/d). Additionally, cheese consumption was linearly associated with a lower risk of major adverse coronary events in women. There was no significant association between cream and the risk of any cardiovascular outcome. We observed no clear associations between any of the dairy products and stroke risk. However, after excluding potential misreporters, high intake of non-fermented milk was associated with a decreased risk of ischaemic stroke but an increased risk of haemorrhagic stroke.

In a systematic review of six observational studies, non-fermented milk was not associated with CHD^(11)^. In the meta-analysis, ‘high *v*. low’ intakes for each cohort study were compared and the dose–response analysis was per 200 g/d. Such an approach has been critiqued because it does not take the level of intake into account and extrapolates results from studies with small exposure ranges to cover larger ranges. In another systematic review, the heterogeneity was too large to be able to perform a meta-analysis^(21)^. In addition, meta-analyses of nutritional observational studies are challenging with additional aspects and may, as a result, have increased variability and reduced possibility to detect real effects^(22)^. In fact, the dairy intake levels vary, and few studies have examined the association between very high intakes of non-fermented milk and CVD risk. In line with our findings of a higher risk of CHD only among very high consumers of non-fermented milk, 52 % higher risk of coronary events for the high consumers of non-fermented dairy products (median intakes: 907 g/d) compared with the low consumers (0–258 g/d) was observed in a cohort of men in Finland^(23)^. There was no association between non-fermented milk and total stroke in this study. Similarly, a meta-analysis has reported no significant association between non-fermented milk and the risk of stroke^(24)^. Interestingly, we observed, especially after excluding potential misreporters, a protective association between non-fermented milk and ischaemic stroke but a positive association with risk of haemorrhagic stroke (i.e. subarachnoid and intracerebral stroke). In another large Swedish cohort study, high intake of non-fermented milk was also positively associated with haemorrhagic stroke, while there was no association with ischaemic stroke^(25)^. In addition, in the European EPIC cohort with 12·7 years of follow-up (where MDCS is one of the centres), a protective association was found with ischaemic stroke and a marginally higher risk of haemorrhagic stroke, especially after excluding the first 4 years of follow-up^(26)^.

In a systematic review and meta-analysis by Fontecha *et al*.^(6)^, fermented milk was associated with a lower risk of CVD and related biomarkers, such as LDL-cholesterol and blood pressure. Moreover, a multinational cohort study from five continents in twenty-one countries reported lower risk of major CVD and composite outcomes with higher intake of fermented milk, specifically yogurt (median intakes of > 1·5 *v*. 0 g/d)^(10)^. These findings are consistent with the present results; moderate intakes of fermented milk (100–300 g/d) were significantly associated with a lower risk of coronary events compared with low intakes. However, there was no reduced risk among higher consumers of fermented milk (>300 g/d) for major adverse coronary events or any of the other outcomes. The lack of an association in the higher group may be explained by lower power (i.e. few numbers of individuals) in the higher group. However, a U-shaped association has previously been shown between fermented milk and cardiovascular mortality in a similar Swedish population^(27)^. In a meta-analysis of fifteen cohort studies, fermented milk intake (average = 200 g/d) was significantly associated with a 20 % lower risk of stroke^(24)^.

In the current study, we observed a lower risk of CHD associated with cheese intake among women, but no association was observed for stroke. In a meta-analysis of thirty-one cohort studies, cheese intake was inversely associated with the risk of CHD^(3)^. Similarly, another meta-analysis of fifteen cohort studies has reported an inverse association between cheese intake and the risk of CVD, CHD and stroke, although the association was nonlinear^(28)^. Other meta-analyses and systematic reviews have also reported a lower risk of coronary events^(11)^ and stroke^(24)^ associated with cheese intake.

Non-fermented milk is a major source of d-galactose, which has been hypothesised to have a detrimental effect on CVD. For example, when d-galactose was given to laboratory animals subcutaneously, they were predisposed to premature ageing caused by oxidative stress and chronic inflammation^(29,30)^. However, the different results for non-fermented and fermented milk on major adverse coronary events risk are not easily explained. We have previously found that non-fermented milk intake was positively associated with plasma leptin levels, which has linked to endothelial dysfunction^(31)^, and inversely associated with HDL-cholesterol levels^(32)^. Several potential biological mechanisms can explain the association between fermented dairy products and CVD, for example, the bacteria found in fermented milk and cheese. Mechanistically, bacteria are suggested to produce SCFA and ferment indigestible carbohydrates, which inhibit the synthesis of cholesterol^(33)^, and could thereby lower the blood cholesterol level. The bacteria in the large intestine can bind cholesterol to bile acids to form cholesterol-bile acid complexes, which are excreted in faeces; the reduced bile acid circulation inhibits the take-up of cholesterol from the circulation into the liver^(33)^. In a human trial, the consumption of 200 ml fermented milk daily reduced LDL-cholesterol concentrations after 3 months; however, after 6 months, there were no difference in LDL-cholesterol compared with the control group^(34)^. In addition, the confounding structure for non-fermented and fermented milk differed. For example, while high intake non-fermented milk was associated with lower educational level, high intake of fermented milk was associated with higher educational level. Although we were able to adjust for multiple potential confounding, residual confounding may still be present.

In the present study, both moderate (0–20 g/d) and higher (>50 g/d) butter intakes were associated with a reduced risk of coronary events compared with zero consumers, while no significant association was observed with stroke risk. A systematic review and meta-analysis found no significant association between butter consumption with the risk of CHD or stroke^(35)^. Cream consumption was not associated with risk of major adverse coronary events or stroke in the current study. In line with our results, other studies^(24)^ have reported no association between cream and the risk of stroke. However, cream intake was inversely associated with the risk of ischaemic stroke and intracerebral haemorrhage, but not subarachnoid haemorrhage, in a study of Finnish male smokers^(36)^. Relatively few studies have examined the health effects of these high-fat dairy products and more studies examining the relation to CVD risk are needed. In a meta-analysis of eighteen observational studies, higher levels of biomarkers of dairy fat measured in blood (i.e. 15:0 and 17:0) were associated with lower risk of CVD^(37)^.

Similar results were observed regardless of whether the intakes were categorised based on absolute intakes (i.e. g/d) or based on energy-adjusted variables (using the residual model or the nutrient density model). When using energy-adjusted variables, the intakes were standardised according to the median energy intake in this population (i.e. evaluated at an isoenergetic level) and therefore fully independent on body size, metabolic efficiency and physical activity, which are all determinants of energy intake^(38)^. The slight differences in results when investigating linear associations between intake of butter and major adverse coronary events could be due to the residual and nutrient density approaches having more power to detect associations than the standard multivariate model when the exposure variables are categorised^(38,39)^. Most studies investigating the association between dairy consumption and health effects use absolute intake for categorisation and adjust for energy intake in the model (i.e. standard multivariable model). Therefore, we decided to use the standard multivariate model as the main model since it is easier to interpret than the residual model, and unlike the nutrient density approach, it also allows us to compare the results to other studies and recommendations.

The strengths of this study include its large population-based cohort design, long-time follow-up and detailed classification of major adverse coronary events (including myocardial infarction, death due to ischaemic health disease, coronary artery bypass graft surgery and percutaneous coronary artery intervention) and stroke from registers. Additionally, the 7-d food records and dietary questionnaire provided detailed information on milk and dairy consumption, which enabled us to analyse non-fermented milk, fermented milk, cheese, butter and cream separately. For example, milk consumed as drink and used in cooked foods was collected in the 7-d food record and milk in coffee, tea, porridge and breakfast cereals was collected in the dietary questionnaire. Most other studies have collected data on milk intake from questionnaires. However, similar intakes have been observed in Swedish studies with data collection during the same period although different diet assessment methods were used^(27)^. We also have information on other lifestyle and dietary factors, which enabled us to adjust for multiple potential confounders. However, there were some limitations. The dietary data were self-reported, which are prone to recall bias^(40)^, and might have affected our results due to the misclassification of food intakes. Moreover, the study population included restricts the generalisability of our findings to the external population. Finally, the analytic sample may be different from the target sample. While representative samples are necessary for estimating prevalence or incidence, this is not the case when the study aims to examine the association between exposure and outcome^(41)^.

In conclusion, a very high intake of non-fermented milk (>1000 g/d) was associated with an increased risk of major adverse coronary events, while no significant association was observed with stroke. In contrast, moderate intake of fermented milk, butter and cheese was associated with lower risk of major adverse coronary events, whereas no significant association was observed for cream. These results highlight the importance of studying dairy products separately, and future studies should also incorporate metabolomics data to investigate the differences more in detail. The consumption of milk and other dairy products is high in Sweden. Thus, even small harmful or beneficial health effects would have a large impact on the population and community, and future studies in populations with high intakes are encouraged.

## Supplementary material

For supplementary material accompanying this paper visit https://doi.org/10.1017/S0007114523001939. click here to view supplementary material
