# Supplementary material for: High consumption of dairy products and risk of major adverse coronary events and stroke in a Swedish population
Source: Br J Nutr. 2023 Sep 6;131(3):500–11. doi: 10.1017/S0007114523001939 (PMC10784127; doi:10.1017/S0007114523001939)
Supplement: Supplementary file 1 [file S0007114523001939sup001.docx]

**Supplemental table 1**. Association (hazard ratios and 95% CI) between cream intakes and risk of major adverse coronary events and stroke

| **Outcomes** |  | **Cream (g/day)** | | | | | | | |
| --- | --- | --- | --- | --- | --- | --- | --- | --- | --- |
|  | **0-10 (n=12,483)** | | **10-20 (n=6,929)** | **20-30 (n=3,405)** | **30-40 (n=1,624)** | **40-50 (n=831)** | **>50 (n=918)** | **Per 10g/day** | **P-trend** |
| **Major adverse**  **coronary events** | 1,765 | | 926 | 447 | 231 | 123 | 141 |  |  |
| Person-years | 237,845 | | 133,177 | 65,745 | 31,370 | 15,998 | 17,496 |  |  |
| Model 1 | 1.00 | | 0.92 (0.85–1.00) | 0.85 (0.76–0.94) | 0.88 (0.77–1.01) | 0.87 (0.72–1.04) | 0.87 (0.73–1.04) | 0.97 (0.95-0.99) | 0.008 |
| Model 2 | 1.00 | | 0.97 (0.90–1.06) | 0.90 (0.81–1.00) | 0.93 (0.81–1.07) | 0.90 (0.75–1.08) | 0.90 (0.75–1.07) | 0.98 (0.96-1.00) | 0.08 |
| Model 3 | 1.00 | | 0.99 (0.91–1.07) | 0.91 (0.82–1.02) | 0.95 (0.83–1.10) | 0.91 (0.76–1.10) | 0.92 (0.77–1.10) | 0.99 (0.97-1.01) | 0.19 |
| **Coronary events** | 1,411 | | 734 | 360 | 177 | 104 | 119 |  |  |
| Person-years | 241,620 | | 135,206 | 66,547 | 31,969 | 16,199 | 17,736 |  |  |
| Model 1 | 1.00 | | 0.91 (0.83–0.99) | 0.85 (0.76–0.95) | 0.83 (0.71–0.97) | 0.92 (0.75–1.12) | 0.92 (0.76–1.12) | 0.98 (0.95-1.00) | 0.04 |
| Model 2 | 1.00 | | 0.95 (0.88–1.05) | 0.90 (0.80–1.02) | 0.88 (0.75–1.03) | 0.95 (0.77–1.16) | 0.94 (0.78–1.14) | 0.99 (0.96-1.01) | 0.20 |
| Model 3 | 1.00 | | 0.98 (0.89–1.07) | 0.92 (0.81–1.03) | 0.90 (0.77–1.06) | 0.96 (0.78–1.17) | 0.97 (0.80–1.17) | 0.99 (0.97-1.01) | 0.36 |
| **Total stroke** | 1,233 | | 698 | 326 | 183 | 103 | 100 |  |  |
| Person-years | 241,770 | | 135,397 | 66,571 | 31,962 | 16,004 | 17,924 |  |  |
| Model 1 | 1.00 | | 0.97 (0.88–1.05) | 0.87 (0.77–0.98) | 0.99 (0.84–1.15) | 1.09 (0.89–1.34) | 0.91 (0.74–1.12) | 0.99 (0.96-1.01) | 0.33 |
| Model 2 | 1.00 | | 1.00 (0.91–1.10) | 0.90 (0.79–1.02) | 1.02 (0.87–1.19) | 1.13 (0.92–1.38) | 0.93 (0.76–1.15) | 1.00 (0.97-1.02) | 0.68 |
| Model 3 | 1.00 | | 1.01 (0.92–1.11) | 0.91 (0.80–1.03) | 1.03 (0.88–1.21) | 1.13 (0.92–1.39) | 0.95 (0.77–1.17) | 1.00 (0.97-1.02) | 0.82 |
| **Ischemic stroke** | 1,016 | | 575 | 249 | 152 | 83 | 80 |  |  |
| Model 1 | 1.00 | | 0.96 (0.87–1.07) | 0.80 (0.70–0.92) | 0.99 (0.83–1.17) | 1.06 (0.84–1.33) | 0.88 (0.70–1.11) | 0.98 (0.95-1.01) | 0.15 |
| Model 2 | 1.00 | | 1.00 (0.90–1.10) | 0.83 (0.72–0.95) | 1.02 (0.86–1.21) | 1.09 (0.87–1.37) | 0.90 (0.71–1.13) | 0.99 (0.96-1.01) | 0.34 |
| Model 3 | 1.00 | | 1.01 (0.91–1.12) | 0.84 (0.73–0.96) | 1.03 (0.87–1.23) | 1.10 (0.88–1.38) | 0.92 (0.73–1.16) | 0.99 (0.96-1.02) | 0.47 |
| **Hemorrhagic stroke** | 196 | | 113 | 71 | 28 | 19 | 18 |  |  |
| Model 1 | 1.00 | | 0.99 (0.78-1.25) | 1.21 (0.92-1.59) | 0.96 (0.65-1.44) | 1.30 (0.81-2.10) | 1.06 (0.65-1.73) | 1.02 (0.97-1.08) | 0.46 |
| Model 2 | 1.00 | | 1.03 (0.82-1.31) | 1.27 (0.97-1.68) | 1.03 (0.69-1.54) | 1.35 (0.84-2.18) | 1.09 (0.66-1.78) | 1.03 (0.97-1.09) | 0.32 |
| Model 3 | 1.00 | | 1.03 (0.81-1.30) | 1.27 (0.96-1.68) | 1.02 (0.68-1.53) | 1.35 (0.84-2.18) | 1.08 (0.66-1.77) | 1.03 (0.97-1.09) | 0.33 |

Model 1: adjusted for age, sex, diet assessment method, season, and energy. Model 2: adjusted for age, sex, diet assessment method, season, energy, alcohol, smoking, education, physical activity, fiber, vegetable and fruits, meat, soft drinks, and coffee. Model 3: adjusted for age, sex, diet assessment method, season, energy, alcohol, smoking, education, physical activity, fiber, vegetable and fruits, meat, soft drinks, coffee and BMI

**Supplemental table 2**. Association (hazard ratios and 95% CI) between butter intakes and risk of major adverse coronary events and stroke

| **Outcomes** |  | | **Butter (g/day)** | | | | | | | |
| --- | --- | --- | --- | --- | --- | --- | --- | --- | --- | --- |
|  | **0**  **(n=14,826)** | **0-10**  **(n=3,671)** | | **10-20**  **(n=2,185)** | **20-30**  **(n=1,780)** | **30-40**  **(n=1,349)** | **40-50**  **(n=768)** | **>50**  **(n=1,611)** | **Per 10g/day** | **P-trend** |
| **Major adverse coronary events** | 2,185 | 407 | | 253 | 222 | 188 | 124 | 254 |  |  |
| Person-years | 283,933 | 72,432 | | 42,160 | 34,140 | 25,196 | 14,460 | 29,311 |  |  |
| Model 1 | 1.00 | 0.83 (0.75–0.92) | | 0.86 (0.75–0.98) | 0.91 (0.79–1.05) | 0.96 (0.82–1.11) | 1.02 (0.85–1.22) | 0.94 (0.82–1.07) | 1.00 (0.98-1.02) | 0.90 |
| Model 2 | 1.00 | 0.88 (0.79–0.97) | | 0.85 (0.75–0.97) | 0.88 (0.77–1.01) | 0.90 (0.77–1.05) | 0.94 (0.78–1.13) | 0.84 (0.73–0.96) | 0.98 (0.97-1.00) | 0.04 |
| Model 3 | 1.00 | 0.87 (0.78–0.97) | | 0.86 (0.75–0.98) | 0.89 (0.77–1.02) | 0.91 (0.79–1.06) | 0.98 (0.81–1.17) | 0.85 (0.74–0.98) | 0.99 (0.97-1.00) | 0.08 |
| **Coronary events** | 1,728 | 322 | | 207 | 178 | 166 | 96 | 208 |  |  |
| Person-years | 288,893 | 73,237 | | 42,603 | 34,574 | 25,472 | 14,748 | 29,750 |  |  |
| Model 1 | 1.00 | 0.83 (0.74–0.94) | | 0.90 (0.78–1.04) | 0.93 (0.80–1.09) | 1.08 (0.92–1.27) | 1.00 (0.81–1.23) | 1.00 (0.86–1.16) | 1.01 (0.99-1.03) | 0.32 |
| Model 2 | 1.00 | 0.88 (0.78–1.00) | | 0.89 (0.77–1.03) | 0.89 (0.76–1.04) | 1.00 (0.85–1.18) | 0.91 (0.74–1.12) | 0.87 (0.74–1.01) | 0.99 (0.97-1.01) | 0.20 |
| Model 3 | 1.00 | 0.88 (0.78–0.99) | | 0.90 (0.77–1.04) | 0.90 (0.77–1.05) | 1.02 (0.86–1.19) | 0.94 (0.76–1.16) | 0.88 (0.75–1.03) | 0.99 (0.97-1.01) | 0.31 |
| **Total stroke** | 1490 | 346 | | 229 | 178 | 150 | 79 | 171 |  |  |
| Person-years | 289,588 | 72,799 | | 42,462 | 34,434 | 25,536 | 14,814 | 29,995 |  |  |
| Model 1 | 1.00 | 1.02 (0.90–1.14) | | 1.14 (0.99–1.31) | 1.07 (0.92–1.25) | 1.14 (0.97–1.35) | 0.97 (0.77–1.21) | 1.02 (0.86–1.20) | 1.01 (0.99-1.03) | 0.49 |
| Model 2 | 1.00 | 1.04 (0.93–1.17) | | 1.13 (0.98–1.30) | 1.04 (0.89–1.22) | 1.10 (0.93–1.31) | 0.92 (0.73–1.16) | 0.94 (0.79–1.11) | 0.99 (0.97-1.01) | 0.54 |
| Model 3 | 1.00 | 1.04 (0.93–1.17) | | 1.14 (0.99–1.31) | 1.05 (0.90–1.23) | 1.11 (0.94–1.32) | 0.93 (0.74–1.18) | 0.95 (0.80–1.12) | 1.00 (0.98-1.02) | 0.63 |
| **Ischemic stroke** | 1,209 | 284 | | 183 | 151 | 123 | 58 | 147 |  |  |
| Model 1 | 1.00 | 1.03 (0.91–1.18) | | 1.13 (0.96–1.31) | 1.13 (0.95–1.33) | 1.16 (0.96–1.39) | 0.88 (0.67–1.14) | 1.08 (0.90–1.29) | 1.01 (0.99-1.03) | 0.27 |
| Model 2 | 1.00 | 1.07 (0.94–1.22) | | 1.12 (0.96–1.31) | 1.09 (0.92–1.30) | 1.11 (0.92–1.34) | 0.83 (0.63–1.08) | 0.98 (0.81–1.18) | 1.00 (0.98-1.02) | 0.80 |
| Model 3 | 1.00 | 1.07 (0.93–1.21) | | 1.13 (0.96–1.32) | 1.10 (0.93–1.31) | 1.12 (0.93–1.35) | 0.85 (0.65–1.10) | 0.99 (0.82–1.19) | 1.00 (0.98-1.02) | 0.92 |
| **Hemorrhagic stroke** | 258 | 58 | | 40 | 23 | 24 | 20 | 22 |  |  |
| Model 1 | 1.00 | 0.95 (0.71-1.26) | | 1.12 (0.80-1.56) | 0.78 (0.51-1.19) | 1.05 (0.69-1.59) | 1.40 (0.88-2.22) | 0.75 (0.48-1.18) | 0.98 (0.93-1.03) | 0.40 |
| Model 2 | 1.00 | 0.96 (0.72-1.28) | | 1.11 (0.80-1.56) | 0.77 (0.50-1.18) | 1.02 (0.67-1.57) | 1.37 (0.86-2.18) | 0.73 (0.46-1.16) | 0.97 (0.93-1.03) | 0.30 |
| Model 3 | 1.00 | 0.96 (0.72-1.28) | | 1.11 (0.80-1.56) | 0.76 (0.50-1.18) | 1.02 (0.67-1.56) | 1.36 (0.85-2.16) | 0.73 (0.46-1.15) | 0.97 (0.93-1.02) | 0.29 |

Model 1: adjusted for age, sex, diet assessment method, season, and energy. Model 2: adjusted for age, sex, diet assessment method, season, energy, alcohol, smoking, education, physical activity, fiber, vegetable and fruits, meat, soft drinks, and coffee. Model 3: adjusted for age, sex, diet assessment method, season, energy, alcohol, smoking, education, physical activity, fiber, vegetable and fruits, meat, soft drinks, coffee and BMI

**Supplementary table 3**. Association between consumption of milk and other dairy products and the risk of major coronary events and stroke after excluding energy misreporters and diet changers (N=16,931)

| **Outcomes** |  | | **Non-fermented milk (g/day)** | | | | | | | | |  |
| --- | --- | --- | --- | --- | --- | --- | --- | --- | --- | --- | --- | --- |
|  | 0-200 | 200-400 | | 400-600 | 600-800 | 800-1000 | >1000 | |  | Per 100g/day | P-trend | |
| Major adverse coronary events | 1.00 | 1.03 (0.93–1.14) | | 1.08 (0.96–1.21) | 1.01 (0.84–1.20) | 0.94 (0.70–1.27) | 1.39 (1.05–1.84) | |  | 1.01 (1.00-1.03) | 0.16 | |
| Coronary events | 1.00 | 1.05 (0.94–1.17) | | 1.08 (0.94–1.23) | 0.97 (0.79–1.19) | 0.97 (0.70–1.34) | 1.32 (0.96–1.82) | |  | 1.01 (0.99-1.03) | 0.26 | |
| Total stroke | 1.00 | 0.96 (0.85–1.07) | | 0.93 (0.81–1.07) | 0.83 (0.67–1.04) | 0.92 (0.64–1.31) | 0.85 (0.56–1.30) | |  | 0.99 (0.96-1.01) | 0.20 | |
| Ischemic stroke | 1.00 | 0.89 (0.78-1.01) | | 0.82 (0.70-0.97) | 0.82 (0.65-1.04) | 0.82 (0.55-1.22) | 0.63 (0.37-1.06) | |  | 0.97 (0.95-1.00) | 0.02 | |
| Hemorrhagic stroke | 1.00 | 1.43 (1.08-1.89) | | 1.51 (1.08-2.11) | 0.97 (0.54-1.78) | 1.74 (0.79-3.84) | 2.30 (1.02-5.15) | |  | 1.05 (1.00-1.10) | 0.07 | |
|  | **Fermented milk (g/day)** | | | | | | |  | |  |  | |
|  | 0 | 0-100 | | 100-200 | 200-300 | >300 |  | |  |  |  | |
| Major adverse coronary events | 1.00 | 0.99 (0.89–1.09) | | 0.92 (0.82–1.03) | 0.86 (0.73–1.01) | 0.88 (0.69–1.11) |  | |  | 0.96 (0.92-1.00) | 0.04 | |
| Coronary events | 1.00 | 0.95 (0.84–1.06) | | 0.93 (0.82–1.06) | 0.85 (0.71–1.02) | 0.88 (0.68–1.15) |  | |  | 0.96 (0.91-1.00) | 0.08 | |
| Total stroke | 1.00 | 0.89 (0.78–1.00) | | 0.99 (0.87–1.13) | 1.03 (0.87–1.23) | 0.82 (0.62–1.09) |  | |  | 0.99 (0.95-1.04) | 0.73 | |
| Ischemic stroke | 1.00 | 0.87 (0.76-0.99) | | 0.97 (0.84-1.12) | 1.07 (0.88-1.29) | 0.84 (0.62-1.15) |  | |  | 1.00 (0.95-1.06) | 0.98 | |
| Hemorrhagic stroke | 1.00 | 0.93 (0.69-1.25) | | 1.06 (0.78-1.44) | 0.92 (0.59-1.44) | 0.70 (0.34-1.46) |  | |  | 0.95 (0.85-1.07) | 0.43 | |
|  | **Cheese (g/day)** | | | | | | |  | |  |  | |
|  | 0-20 | 20-40 | | 40-60 | 60-80 | 80-100 | >100 | |  | Per 10g/day |  | |
| Major adverse coronary events | 1.00 | 0.92 (0.83–1.03) | | 0.88 (0.78–1.00) | 0.93 (0.80–1.09) | 0.79 (0.64–0.97) | 0.88 (0.70–1.11) | |  | 0.99 (0.97-1.01) | 0.20 | |
| Coronary events | 1.00 | 0.87 (0.77–0.98) | | 0.86 (0.75–0.98) | 0.90 (0.75–1.07) | 0.82 (0.65–1.03) | 0.86 (0.66–1.11) | |  | 0.99 (0.97-1.01) | 0.20 | |
| Total stroke | 1.00 | 0.87 (0.77–0.99) | | 0.86 (0.74–0.99) | 0.86 (0.72–1.04) | 0.85 (0.67–1.08) | 0.92 (0.70–1.21) | |  | 0.98 (0.97-1.00) | 0.09 | |
| Ischemic stroke | 1.00 | 0.82 (0.72-0.95) | | 0.86 (0.73-1.00) | 0.84 (0.68-1.03) | 0.80 (0.61-1.05) | 0.92 (0.68-1.24) | |  | 0.98 (0.96-1.00) | 0.09 | |
| Hemorrhagic stroke | 1.00 | 1.18 (0.86-1.62) | | 0.91 (0.63-1.32) | 0.99 (0.63-1.57) | 1.04 (0.58-1.85) | 0.94 (0.48-1.87) | |  | 0.99 (0.94-1.03) | 0.56 | |
|  |  | **Cream (g/day)** | | | | | |  | |  |  | |
|  | 0-10 | 10-20 | | 20-30 | 30-40 | 40-50 | >50 | |  |  |  | |
| Major adverse coronary events | 1.00 | 0.99 (0.89–1.09) | | 0.92 (0.81–1.04) | 0.96 (0.81–1.13) | 0.93 (0.75–1.17) | 1.00 (0.82–1.23) | |  | 1.00 (0.97-1.02) | 0.70 | |
| Coronary events | 1.00 | 1.00 (0.89–1.12) | | 0.93 (80–1.07) | 0.90 (0.74–1.08) | 0.98 (0.77–1.24) | 1.10 (0.88–1.37) | |  | 1.00 (0.97-1.03) | 1.00 | |
| Total stroke | 1.00 | 0.94 (0.83–1.06) | | 0.92 (0.79–1.06) | 1.09 (0.90–1.31) | 1.14 (0.90–1.45) | 1.02 (0.80–1.30) | |  | 1.01 (0.98-1.04) | 0.37 | |
| Ischemic stroke | 1.00 | 0.95 (0.83-1.08) | | 0.86 (0.73-1.02) | 1.13 (0.92-1.38) | 1.15 (0.89-1.50) | 1.04 (0.80-1.36) | |  | 1.01 (0.98-1.05) | 0.40 | |
| Hemorrhagic stroke | 1.00 | 0.89 (0.66-1.19) | | 1.17 (0.84-1.62) | 0.87 (0.53-1.42) | 1.08 (0.59-1.97) | 0.93 (0.51-1.71) | |  | 1.01 (0.94-1.08) | 0.78 | |
|  | **Butter (g/day)** | | | | | | | | |  |  | |
|  | 0 | 0-10 | | 10-20 | 20-30 | 30-40 | 40-50 | | >50 |  |  | |
| Major adverse coronary events | 1.00 | 0.90 (0.79–1.03) | | 0.90 (0.77–1.06) | 0.91 (0.77–1.08) | 0.97 (0.81–1.15) | 1.12 (0.91–1.37) | | 0.89  (0.76–1.05) | 1.00 (0.98-1.02) | 0.87 | |
| Coronary events | 1.00 | 0.91 (0.78–1.05) | | 0.94 (0.79–1.12) | 0.94 (0.78–1.13) | 1.05 (0.87–1.27) | 1.06 (0.84–1.33) | | 0.89  (0.74–1.06) | 1.00 (0.98-1.02) | 0.88 | |
| Total stroke | 1.00 | 1.04 (0.90–1.21) | | 1.19 (1.01–1.41) | 1.08 (0.89–1.30) | 1.22 (1.00–1.48) | 1.06 (0.82–1.37) | | 0.91  (0.75–1.12) | 1.00 (0.98-1.02) | 0.90 | |
| Ischemic stroke | 1.00 | 1.07 (0.91-1.26) | | 1.24 (1.03-1.49) | 1.13 (0.92-1.39) | 1.27 (1.03-1.58) | 0.95 (0.71-1.28) | | 0.97  (0.78-1.21) | 1.00 (0.98-1.03) | 0.84 | |
| Hemorrhagic stroke | 1.00 | 0.94 (0.66-1.34) | | 0.92 (0.60-1.41) | 0.79 (0.48-1.28) | 0.88 (0.52-1.48) | 1.53 (0.93-2.53) | | 0.64 (0.37-1.12) | 0.97 (0.91-1.03) | 0.35 | |

Adjusted for age, sex, assessment method, season, energy, alcohol, smoking, education, physical activity, fiber, vegetable and fruits, meat, soft drinks, coffee, and BMI

**Supplemental table 4**. Association (hazard ratios and 95% CI) between consumption of milk and other dairy products and the risk of major coronary events and stroke using the residual model for energy adjustments

| **Outcomes** |  | | **Non-fermented milk (g/day)** | | | | | | |  |
| --- | --- | --- | --- | --- | --- | --- | --- | --- | --- | --- |
|  | 0-200 | 200-400 | | 400-600 | 600-800 | 800-1000 | >1000 |  | P-trend | |
| Major adverse coronary events | 1.00 | 1.01 (0.93-1.09) | | 1.08 (0.98-1.20) | 1.07 (0.91-1.26) | 1.02 (0.79-1.31) | 1.58 (1.21-2.06) |  | 0.01 | |
| Coronary events | 1.00 | 0.99 (0.91-1.08) | | 1.06 (0.95-1.19) | 1.07 (0.89-1.28) | 1.00 (0.76-1.33) | 1.51 (1.12-2.04) |  | 0.06 | |
| Total stroke | 1.00 | 0.92 (0.84-1.01) | | 0.97 (0.86-1.09) | 0.94 (0.76-1.15) | 1.18 (0.88-1.57) | 0.95 (0.63-1.45) |  | 0.71 | |
| Ischemic stroke | 1.00 | 0.89 (0.81-0.99) | | 0.93 (0.82-1.06) | 0.93 (0.75-1.16) | 1.14 (0.83-1.57) | 0.83 (0.51-1.34) |  | 0.30 | |
| Hemorrhagic stroke | 1.00 | 1.11 (0.89-1.39) | | 1.12 (0.85-1.49) | 1.03 (0.62-1.70) | 1.47 (0.75-2.91) | 1.46 (0.59-3.61) |  | 0.21 | |
|  | **Fermented milk (g/day)** | | | | | | |  |  | |
|  | 0 | 0-100 | | 100-200 | 200-300 | >300 |  |  |  | |
| Major adverse coronary events | 1.00 | 0.97 (0.89-1.05) | | 0.91 (0.83-0.99) | 0.82 (0.72-0.94) | 0.99 (0.84-1.18) |  |  | 0.01 | |
| Coronary events | 1.00 | 0.99 (0.90-1.08) | | 0.93 (0.84-1.03) | 0.82 (0.71-0.96) | 1.09 (0.90-1.31) |  |  | 0.13 | |
| Total stroke | 1.00 | 0.90 (0.81-0.99) | | 0.95 (0.86-1.06) | 0.95 (0.82-1.09) | 0.84 (0.68-1.04) |  |  | 0.15 | |
| Ischemic stroke | 1.00 | 0.89 (0.80-1.00) | | 0.95 (0.85-1.07) | 0.98 (0.84-1.15) | 0.86 (0.68-1.09) |  |  | 0.34 | |
| Hemorrhagic stroke | 1.00 | 0.93 (0.73-1.18) | | 1.00 (0.78-1.29) | 0.85 (0.59-1.23) | 0.78 (0.46-1.33) |  |  | 0.38 | |
|  | **Cheese (g/day)** | | | | | | |  |  | |
|  | 0-20 | 20-40 | | 40-60 | 60-80 | 80-100 | >100 |  |  | |
| Major adverse coronary events | 1.00 | 1.00 (0.92-1.10) | | 0.93 (0.84-1.03) | 0.92 (0.81-1.05) | 0.90 (0.74-1.07) | 0.90 (0.73-1.11) |  | 0.04 | |
| Coronary events | 1.00 | 0.96 (0.87-1.06) | | 0.91 (0.81-1.02) | 0.92 (0.80-1.06) | 0.86 (0.70-1.06) | 0.84 (0.66-1.06) |  | 0.03 | |
| Total stroke | 1.00 | 0.88 (0.79-0.97) | | 0.91 (0.81-1.02) | 0.93 (0.80-1.08) | 0.86 (0.70-1.07) | 0.87 (0.69-1.11) |  | 0.22 | |
| Ischemic stroke | 1.00 | 0.86 (0.77-0.97) | | 0.90 (0.79-1.02) | 0.92 (0.78-1.08) | 0.81 (0.64-1.03) | 0.89 (0.68-1.16) |  | 0.18 | |
| Hemorrhagic stroke | 1.00 | 0.89 (0.68-1.16) | | 0.97 (0.73-1.29) | 1.05 (0.74-1.48) | 0.95 (0.58-1.58) | 0.78 (0.42-1.44) |  | 0.93 | |
|  |  | **Cream (g/day)** | | | | | |  |  | |
|  | 0-10 | 10-20 | | 20-30 | 30-40 | 40-50 | >50 |  |  | |
| Major adverse coronary events | 1.00 | 0.99 (0.91-1.07) | | 0.97 (0.87-1.09) | 0.94 (0.80-1.11) | 0.73 (0.57-0.93) | 1.00 (0.81-1.23) |  | 0.11 | |
| Coronary events | 1.00 | 0.97 (0.88-1.07) | | 0.95 (0.84-1.08) | 0.93 (0.78-1.12) | 0.75 (0.58-0.98) | 1.06 (0.85-1.33) |  | 0.22 | |
| Total stroke | 1.00 | 0.98 (0.89-1.09) | | 0.90 (0.78-1.03) | 1.06 (0.89-1.27) | 0.78 (0.59-1.03) | 1.14 (0.88-1.41) |  | 0.61 | |
| Ischemic stroke | 1.00 | 0.92 (0.83-1.03) | | 0.87 (0.75-1.01) | 1.03 (0.84-1.26) | 0.80 (0.59-1.08) | 1.06 (0.82-1.38) |  | 0.31 | |
| Hemorrhagic stroke | 1.00 | 1.31 (1.05-1.65) | | 1.08 (0.78-1.49) | 1.26 (0.82-1.93) | 0.70 (0.33-1.48) | 1.29 (0.75-2.23) |  | 0.36 | |
|  | **Butter (g/day)** | | | | | | | |  | |
|  | 0 | 0-10 | | 10-20 | 20-30 | 30-40 | 40-50 | >50 |  | |
| Major adverse coronary events | 1.00 | 0.88 (0.79-0.99) | | 0.87 (0.77-0.99) | 0.87 (0.77-0.99) | 0.94 (0.82-1.09) | 0.83 (0.69-1.00) | 0.90 (0.77-1.04) | 0.007 | |
| Coronary events | 1.00 | 0.87 (0.77-0.99) | | 0.92 (0.80-1.06) | 0.91 (0.79-1.05) | 1.01 (0.87-1.18) | 0.85 (0.69-1.04) | 0.89 (0.75-1.05) | 0.08 | |
| Total stroke | 1.00 | 1.06 (0.93-1.20) | | 0.95 (0.82-1.10) | 1.17 (1.03-1.34) | 1.10 (0.94-1.29) | 1.11 (0.90-1.35) | 0.90 (0.74-1.08) | 0.49 | |
| Ischemic stroke | 1.00 | 1.06 (0.91-1.22) | | 0.92 (0.78-1.08) | 1.27 (1.10-1.46) | 1.09 (0.91-1.30) | 1.07 (0.85-1.35) | 0.95 (0.78-1.17) | 0.29 | |
| Hemorrhagic stroke | 1.00 | 0.97 (0.70-1.34) | | 1.08 (0.78-1.34) | 0.75 (0.51-1.10) | 1.14 (0.78-1.66) | 1.35 (0.87-2.11) | 0.60 (0.34-1.04) | 0.52 | |

Adjusted for age, sex, assessment method, season, energy, alcohol, smoking, education, physical activity, fiber, vegetable and fruits, meat, soft drinks, coffee, and BMI

**Supplemental table 5**. Association (hazard ratios and 95% CI) between consumption of milk and other dairy products and the risk of major coronary events and stroke using the nutrient density method for energy adjustments

| **Outcomes** | **Non-fermented milk (g/day)** | | | | | | | |
| --- | --- | --- | --- | --- | --- | --- | --- | --- |
|  | 0-200 | 200-400 | 400-600 | 600-800 | 800-1000 | >1000 |  | P-trend |
| Major adverse coronary events | 1.00 | 1.00 (0.93-1.09) | 1.06 (0.96-1.16) | 1.04 (0.90-1.20) | 1.21 (0.98-1.50) | 1.47 (1.10-1.97) |  | 0.02 |
| Coronary events | 1.00 | 1.02 (0.93-1.11) | 1.04 (0.93-1.15) | 1.03 (0.88-1.20) | 1.17 (0.93-1.48) | 1.50 (1.09-2.04) |  | 0.06 |
| Total stroke | 1.00 | 0.93 (0.85-1.02) | 0.97 (0.86-1.08) | 0.96 (0.81-1.14) | 0.94 (0.72-1.24) | 1.15 (0.79-1.69) |  | 0.66 |
| Ischemic stroke | 1.00 | 0.88 (0.80-0.98) | 0.95 (0.84-1.07) | 0.84 (0.69-1.02) | 0.95 (0.71-1.27) | 1.07 (0.70-1.64) |  | 0.19 |
| Hemorrhagic stroke | 1.00 | 1.19 (0.95-1.49) | 0.99 (0.74-1.32) | 1.67 (1.17-2.37) | 0.84 (0.39-1.79) | 1.45 (0.59-3.57) |  | 0.14 |
|  | **Fermented milk (g/day)** | | | | | |  |  |
|  | 0 | 0-100 | 100-200 | 200-300 | >300 |  |  |  |
| Major adverse coronary events | 1.00 | 0.98 (0.90-1.06) | 0.88 (0.80-0.96) | 0.91 (0.80-1.03) | 0.89 (0.75-1.05) |  |  | 0.008 |
| Coronary events | 1.00 | 1.00 (0.91-1.09) | 0.90 (0.81-1.00) | 0.94 (0.81-1.07) | 0.94 (0.78-1.13) |  |  | 0.09 |
| Total stroke | 1.00 | 0.92 (0.83-1.01) | 0.92 (0.82-1.02) | 0.95 (0.82-1.09) | 0.89 (0.73-1.07) |  |  | 0.12 |
| Ischemic stroke | 1.00 | 0.91 (0.82-1.02) | 0.92 (0.82-1.04) | 0.97 (0.83-1.13) | 0.91 (0.74-1.12) |  |  | 0.28 |
| Hemorrhagic stroke | 1.00 | 0.97 (0.76-1.22) | 0.92 (0.70-1.20) | 0.90 (0.64-1.28) | 0.86 (0.54-1.36) |  |  | 0.38 |
|  | **Cheese (g/day)** | | | | | |  |  |
|  | 0-20 | 20-40 | 40-60 | 60-80 | 80-100 | >100 |  |  |
| Major adverse coronary events | 1.00 | 0.99 (0.91-1.07) | 0.93 (0.84-1.03) | 0.87 (0.77-0.99) | 0.94 (0.79-1.11) | 0.88 (0.71-1.10) |  | 0.03 |
| Coronary events | 1.00 | 0.94 (0.86-1.04) | 0.91 (0.82-1.01) | 0.85 (0.74-0.98) | 0.93 (0.77-1.13) | 0.80 (0.63-1.03) |  | 0.02 |
| Total stroke | 1.00 | 0.85 (0.76-0.94) | 0.88 (0.79-0.99) | 0.92 (0.79-1.06) | 0.97 (0.80-1.17) | 0.79 (0.62-1.02) |  | 0.24 |
| Ischemic stroke | 1.00 | 0.83 (0.74-0.93) | 0.86 (0.76-0.98) | 0.90 (0.77-1.05) | 0.93 (0.75-1.15) | 0.79 (0.59-1.05) |  | 0.17 |
| Hemorrhagic stroke | 1.00 | 0.92 (0.72-1.19) | 0.98 (0.74-1.30) | 1.05 (0.74-1.48) | 1.08 (0.68-1.71) | 0.90 (0.50-1.63) |  | 0.78 |
|  |  | **Cream (g/day)** | | | | |  |  |
|  | 0-10 | 10-20 | 20-30 | 30-40 | 40-50 | >50 |  |  |
| Major adverse coronary events | 1.00 | 0.93 (0.86-1.01) | 1.03 (0.93-1.14) | 0.94 (0.82-1.08) | 0.84 (0.69-1.02) | 0.91 (0.75-1.11) |  | 0.14 |
| Coronary events | 1.00 | 0.92 (0.84-1.01) | 1.01 (0.90-1.13) | 0.91 (0.78-1.07) | 0.86 (0.69-1.07) | 0.96 (0.78-1.18) |  | 0.23 |
| Total stroke | 1.00 | 0.97 (0.89-1.07) | 0.98 (0.87-1.10) | 0.93 (0.79-1.09) | 0.90 (0.72-1.12) | 1.07 (0.87-1.32) |  | 0.62 |
| Ischemic stroke | 1.00 | 0.95 (0.86-1.06) | 0.95 (0.83-1.08) | 0.92 (0.77-1.10) | 0.84 (0.65-1.08) | 1.08 (0.86-1.35) |  | 0.41 |
| Hemorrhagic stroke | 1.00 | 1.07 (0.85-1.35) | 1.18 (0.90-1.56) | 0.98 (0.66-1.46) | 1.25 (0.77-2.03) | 0.95 (0.55-1.64) |  | 0.58 |
|  | **Butter (g/day)** | | | | | | |  |
|  | 0 | 0-10 | 10-20 | 20-30 | 30-40 | 40-50 | >50 |  |
| Major adverse coronary events | 1.00 | 0.86 (0.77-0.96) | 0.88 (0.77-1.00) | 0.88 (0.77-1.00) | 0.97 (0.84-1.11) | 0.81 (0.67-0.96) | 0.92 (0.78-1.07) | 0.01 |
| Coronary events | 1.00 | 0.88 (0.78-1.00) | 0.87 (0.75-1.01) | 0.94 (0.81-1.08) | 1.05 (0.90-1.21) | 0.81 (0.66-0.99) | 0.92 (0.78-1.10) | 0.12 |
| Total stroke | 1.00 | 1.06 (0.94-1.20) | 0.97 (0.83-1.12) | 1.19 (1.03-1.37) | 1.05 (0.90-1.24) | 1.11 (0.92-1.35) | 0.87 (0.71-1.06) | 0.68 |
| Ischemic stroke | 1.00 | 1.07 (0.94-1.23) | 0.97 (0.82-1.14) | 1.24 (1.07-1.45) | 1.05 (0.88-1.26) | 1.07 (0.86-1.33) | 0.95 (0.77-1.17) | 0.43 |
| Hemorrhagic stroke | 1.00 | 1.02 (0.76-1.37) | 0.93 (0.65-1.33) | 0.91 (0.62-1.32) | 1.05 (0.71-1.55) | 1.35 (0.88-2.07) | 0.50 (0.27-0.93) | 0.43 |

Adjusted for age, sex, assessment method, season, energy, alcohol, smoking, education, physical activity, fiber, vegetable and fruits, meat, soft drinks, coffee, and BMI

**Supplemental table 6**. Interaction between milk and dairy consumption on major coronary events and stroke

|  | Non-fermented milk | Fermented milk | Cheese | Cream | Butter |
| --- | --- | --- | --- | --- | --- |
| **Interaction by sex** |  |  |  |  |  |
| Major adverse coronary events | 0.47 | 0.24 | 0.04 | 0.83 | 0.90 |
| Coronary events | 0.21 | 0.21 | 0.02 | 0.44 | 0.90 |
| Total stroke | 0.04 | 0.92 | 0.92 | 0.58 | 0.31 |
| Ischemic stroke | 0.12 | 0.42 | 0.35 | 0.32 | 0.27 |
| Hemorrhagic stroke | 0.14 | 0.07 | 0.13 | 0.98 | 0.49 |
| **Interaction by BMI** |  |  |  |  |  |
| Major adverse coronary events | 0.33 | 0.40 | 0.63 | 0.98 | 0.82 |
| Coronary events | 0.51 | 0.47 | 0.32 | 0.74 | 0.57 |
| Total stroke | 0.55 | 0.31 | 0.19 | 0.66 | 0.72 |
| Ischemic stroke | 0.99 | 0.36 | 0.32 | 0.48 | 0.71 |
| Hemorrhagic stroke | 0.13 | 0.62 | 0.21 | 0.53 | 0.85 |
| **Interaction by physical activity** |  |  |  |  |  |
| Major adverse coronary events | 0.02 | 0.74 | 0.77 | 0.42 | 0.21 |
| Coronary events | 0.03 | 0.78 | 0.76 | 0.63 | 0.10 |
| Total stroke | 0.40 | 0.09 | 0.20 | 0.04 | 0.11 |
| Ischemic stroke | 0.81 | 0.58 | 0.29 | 0.07 | 0.10 |
| Hemorrhagic stroke | 0.33 | 0.01 | 0.23 | 0.33 | 0.71 |

The values indicate P-values for interaction
